# A Step Forward in Breast Cancer Research: From a Natural-Like Experimental Model to a Preliminary Photothermal Approach

**DOI:** 10.3390/ijms21249681

**Published:** 2020-12-18

**Authors:** Eduardo Costa, Tânia Ferreira-Gonçalves, Miguel Cardoso, João M. P. Coelho, Maria Manuela Gaspar, Pedro Faísca, Lia Ascensão, António S. Cabrita, Catarina Pinto Reis, Isabel V. Figueiredo

**Affiliations:** 1 Pharmacology and Pharmaceutical Care Laboratory, Faculty of Pharmacy, University of Coimbra, Azinhaga de Santa Comba, 3000-548 Coimbra, Portugal; eduardo.leitao.costa@gmail.com (E.C.); isabel.vitoria@netcabo.pt (I.V.F.); 2 Institute of Experimental Pathology, Faculty of Medicine, University of Coimbra, Azinhaga de Santa Comba, 3000-548 Coimbra, Portugal; miguel.cardoso16@gmail.com (M.C.); amscabrita@gmail.com (A.S.C.); 3 iMed.ULisboa– Research Institute for Medicines, Faculdade de Farmácia, Universidade de Lisboa, Av. Prof. Gama Pinto, 1649-003 Lisboa, Portugal; taniag1@ff.ulisboa.pt (T.F.-G.); mgaspar@ff.ulisboa.pt (M.M.G.); 4 Vasco da Gama Research Group (CIVG), Vasco da Gama University School (EUVG), 3020-210 Coimbra, Portugal; 5 Dentistry Area, Faculty of Medicine, University of Coimbra, Azinhaga de Santa Comba, 3000-548 Coimbra, Portugal; 6 Biophysics Institute, Faculty of Medicine, University of Coimbra, Azinhaga de Santa Comba, 3000-548 Coimbra, Portugal; 7 Institute for Clinical and Biomedical Research (iCBR), Faculty of Medicine, University of Coimbra, Azinhaga de Santa Comba, 3000-548 Coimbra, Portugal; 8 Instituto de Biofísica e Engenharia Biomédica, Faculdade de Ciências, Universidade de Lisboa, 1749-016 Lisboa, Portugal; jmcoelho@fc.ul.pt; 9 Faculty of Veterinary Medicine (ULHT)/IGC, 1749-024 Lisboa, Portugal; pedrofaisca76@gmail.com; 10 Centro de Estudos do Ambiente e do Mar (CESAM), Faculdade de Ciências, Campo Grande, Universidade de Lisboa, 1749-016 Lisboa, Portugal; lmpsousa@fc.ul.pt

**Keywords:** breast cancer, experimental model, DMBA, laser photothermal therapy, gold nanoparticles, epigenetic alterations

## Abstract

Breast cancer is one of the most frequently diagnosed malignancies and common causes of cancer death in women. Recent studies suggest that environmental exposures to certain chemicals, such as 7,12-Dimethylbenzanthracene (DMBA), a chemical present in tobacco, may increase the risk of developing breast cancer later in life. The first-line treatments for breast cancer (surgery, chemotherapy or a combination of both) are generally invasive and frequently associated with severe side effects and high comorbidity. Consequently, novel approaches are strongly required to find more natural-like experimental models that better reflect the tumors’ etiology, physiopathology and response to treatments, as well as to find more targeted, efficient and minimally invasive treatments. This study proposes the development and an in deep biological characterization of an experimental model using DMBA-tumor-induction in Sprague-Dawley female rats. Moreover, a photothermal therapy approach using a near-infrared laser coupled with gold nanoparticles was preliminarily assessed. The gold nanoparticles were functionalized with Epidermal Growth Factor, and their physicochemical properties and in vitro effects were characterized. DMBA proved to be a very good and selective inductor of breast cancer, with 100% incidence and inducing an average of 4.7 tumors per animal. Epigenetic analysis showed that tumors classified with worst prognosis were hypomethylated. The tumor-induced rats were then subjected to a preliminary treatment using functionalized gold nanoparticles and its activation by laser (650–900 nm). The treatment outcomes presented very promising alterations in terms of tumor histology, confirming the presence of necrosis in most of the cases. Although this study revealed encouraging results as a breast cancer therapy, it is important to define tumor eligibility and specific efficiency criteria to further assess its application in breast cancer treatment on other species.

## 1. Introduction

Breast cancer is a global public health issue and it is the most frequently diagnosed malignancy among women in the Western world. According to GLOBOCAN (Global Cancer Observatory report) 2018 it is one of the most common causes of cancer death in European and American women. According to the American Cancer Society, the incidence rate increased 0.3% per year from 2012–2016 [1,2,3], while the cancer death rate declined between 1989 and 2017, dropping 40% [3], which might be a consequence of the increased awareness of the population in combination with the access to improved diagnostic and therapeutic options.

Breast cancer involves a heterogeneous group of tumors which present variable prognosis and resistance to therapy [4]. There are different classification systems based on the tumor’s size, histological subtype grade, lymph node status and expression of different genes, proteins and receptors, such as the estrogen receptor (ER), the progesterone receptor (PR) and the human epidermal growth factor receptor 2 (HER2) [4]. Some of those systems distinguish breast tumors into different histopathological and molecular subtypes [5,6,7]. The etiology behind different tumor types is still under analysis. Nevertheless, there are multiple studies associating different tumor classifications and incidence rates per demographic regions with individual features [1,3], such as age, race and genetics (BRCA1 and BRCA2) [8,9], environmental factors [10,11], such as smoking habits, exposure to chemicals and radiation. Attending to the individual features, epigenetic changes including DNA methylation, histone modifiers and readers, chromatin remodelers and microRNAs [12,13] have also been associated with cancer development [12,14]. Among the environmental factors, tobacco smoke has attracted special attention since it has been reported a possible correlation between the increased incidence rate of breast cancer and the tobacco smoke exposure [15,16,17,18,19,20,21,22,23,24,25,26,27,28].

Several treatment options are available for breast cancer, including surgery, radiation and/or chemotherapy. Nonetheless, most of them have significant limitations, so efforts must be gathered to establish new therapeutic approaches, which depend greatly on the access to reliable experimental models that are able to mimic tumors’ etiology, physiopathology and/or response to treatments.

Experimental models are crucial tools to unveil breast cancer features, and to develop and evaluate potential diagnostic and new therapeutic strategies. A vast list of experimental models has been proposed [29,30], including, for instance, in vitro [31,32] and in vivo models. The choice of the most suitable model is not easy, and it depends on the researcher’s preference and background knowledge, on the resources and infrastructures available, on the purpose/focus of the study and on the ethical concerns inherent to the use of biological samples. In vivo models [33,34] keep playing a key role in biomedical and pharmaceutical research. Different models have been proposed with murine being the most frequently used in the European Union [35]. Among those, female rat models of breast cancer present many similarities with women [36,37] in terms of molecular and genetic features, biochemical properties, histology and hormone response, which turn them the most used models [38]. In vivo models include, among others, two big groups: transplanted tumor models, which rely on the transplantation of suspensions containing living cancer cells or solid tumors obtained from donors [39]; and chemically-induced models [5,40,41], which consist on the induction of tumors upon exposure to chemical compounds. Tumor transplanted models are well-established models, typically associated with a more controlled development of more homogeneous and better-defined tumors [39]. However, they require the use of immunocompromised animals, which do not mimic a real scenario and makes these models very expensive. On the other hand, chemically-induced models are cheaper models that result in more heterogeneous tumors with longer latency, whose development is harder to control [29]. However, they are especially useful to understand the impact of certain chemicals, to which people might be exposed to in their routine, on breast cancer initiation, promotion and progression [5,40,41]. The 7,12-Dimethylbenzanthracene (DMBA), a very little water-soluble compound highly lipophilic can be found in a list of chemicals being studied as possible breast cancer initiators and promotors [42]. It is present in cigarette smoke, coal, burned wood, coal tar and gasoline or diesel engines, and can be absorbed through the skin, respiratory and gastrointestinal tract [42,43,44,45]. DMBA has been associated to various immunotoxic, mutagenic, teratogenic and carcinogenic effects and it has been used in animal experimentation as an inducer and promotor of neoplasia, regardless of the route of administration [40,42,44,46]. Despite the existent of a very small number of reports on DMBA-induced breast cancer models, there are no reference values for hematological, urine parameters and epigenetic alterations associated to DMBA breast cancer induced models [47,48,49,50].

As a potential therapy, photothermal therapy (PTT) is emerging as clinically viable and of great interest in superficial cancers’ treatment. It consists of inducing thermal ablation of cancer cells upon their irradiation with light beams [51,52]. Although promising, its effect and use depends greatly on the deepness reached by the light and on the heat generated. Thus, one strategy explored to enhance the photothermal therapeutic effect relies on the use of near-infrared (NIR) radiation, radiation with higher tissue penetration capability [52,53], and on the administration of nanoparticles (NPs) into the tumor. For this purpose, gold nanoparticles (GNPs) are highly valuable due to their tunable optical properties [54,55]. GNPs have a marked Surface Plasmon Resonance (SPR) [56]. When irradiated with light at their maximum absorbance wavelength, GNPs will convert the light energy into heat [54,57], which will then be dissipated and may lead to the destruction of the targeted tissues through a necrosis pathway [57]. In order to improve the GNPs’ specificity towards cancer cells, the addition of coating layers, such as hyaluronic acid (HA) [55,58], has been proposed. HA has been reported as a natural ligand to CD44 receptors overexpressed in certain types of breast cancers [59], and ligands [56,58], such as Epidermal Growth Factor (EGF) [56,58,60], a natural peptide ligand to Epidermal Growth Factor receptors commonly overexpressed in some tumor cells [61].

Herein, breast cancer was chemically induced with DMBA in Sprague-Dawley female rats. The experimental model was fully characterized by assessing the body weight and urine parameters over time, as well as the final hematological/biochemical parameters, histological analysis and 5-Methylcytosine (5mC) quantification of tumors, which is a major form of DNA alteration commonly found in eukaryotic cells [62,63,64,65]. Furthermore, preliminary in vitro and in vivo studies were also carried out to assess the use of EGF-conjugated GNPs coated with a combination of hyaluronic and oleic acids combined with NIR laser irradiation for improvement of photothermal therapy as a treatment modality for breast cancer.

## 2. Results

### 2.1. Experimental Model Characterization

#### 2.1.1. Animals’ Weight

**Figure 1:**
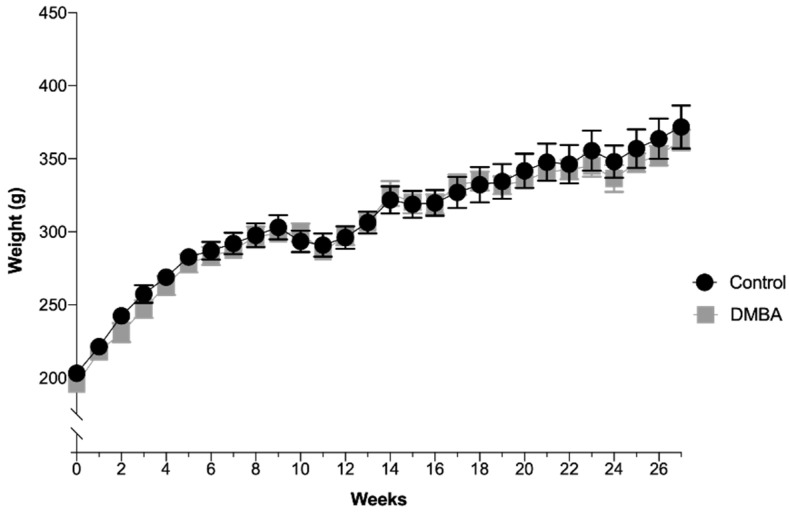
Evolution of body weight over time expressed in grams (Mean ± SEM). DMBA administration occurred in the first week, represented in the graph as week 0, time at which rats were 50 days-old.

The animals’ average body weight per group over time is depicted in Figure 1. There were no significant differences between the two groups during the experimental time, even after DMBA administration. In the control group (*n* = 10), the average weight was 308.1 ± 8.0 g with a minimum value of 203.2 g and a maximum value of 371.7 g. In the test group (DMBA, *n* = 10), the average weight was 304.1 ± 8.0 g with a minimum value of 195.7 g and a maximum value of 362.9 g. All animals were in good state of welfare and there were no signs of suffering, according to animal welfare guidelines.

#### 2.1.2. Urinalysis

Sixty urine samples were collected in each group, ten per month, 120 samples in total. According to different studies [66,67,68], urine samples frozen at −20 °C are stable in the first two years, and their properties do not change.

The results from both control group (*n* = 60) and DMBA group (*n* = 60) are depicted in Table 1. Additionally, the specific gravity mean was 1.022 and 1.018 for the control group and DMBA group, respectively.

No significant differences were identified when comparing the measured urine parameters between the two groups.

#### 2.1.3. Blood Samples

The findings of the blood samples analysis are presented in Table 2. Although visually there were slight differences noticed between the two groups, when attending the statistical analysis, no significant differences (*p* < 0.05) were detected.

#### 2.1.4. Histopathological Classification of the Breast Tumors

**Figure 2:**
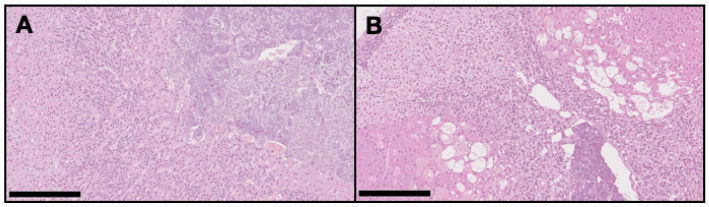
Representative light microscopy images of the adrenal glands in the control group (**A**) with no pathological alterations, and in the DMBA group (**B**) with severe cortical cystic degeneration (H&E, 100×). Scale bar = 250 µm.

**Figure 3:**
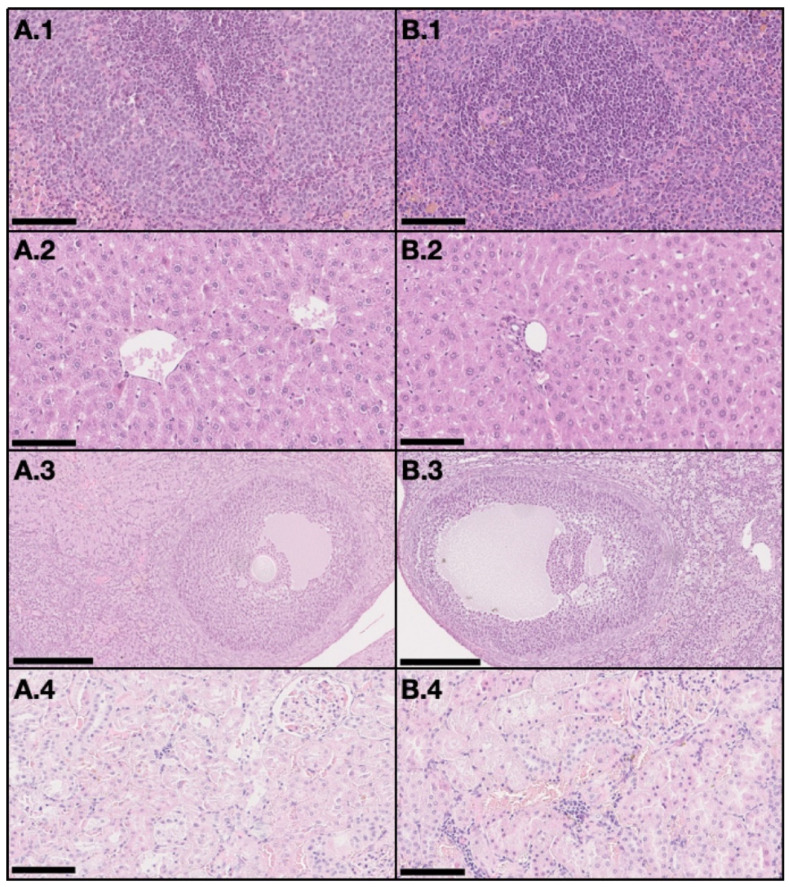
Representative light microscopy images with no signs of toxicity in non-target organs, both in the control group (**A**) and the DMBA group (**B**). Each row represents an organ (1–spleen (200×), 2–liver (200×), 3–ovary (100×), and 4–kidney (200×)) (H&E). Scale bar = 250 µm (A.3 and B.3) and 100 µm (all the others).

On the DMBA group, all animals had at least one mammary tumor (100% incidence), with an average of 4.7 tumors per animal. Nine animals had at least one invasive malignant tumor (90% incidence), with an average of 3.67 invasive tumors per animal. For non-target organs, DBMA-induced lesions were only observed on the adrenal glands with cortical cystic degeneration (Figure 2) in 70% of animals. Kidneys from the animals in the DMBA group presented discrete interstitial mononuclear inflammatory infiltrates that were interpreted as background lesions. Other non-target organs, such as spleen, liver and ovary showed no signs of toxicity when compared to the control group (Figure 3).

##### Breast Tumors Classification

Sixty mammary tumor samples were evaluated and graded. Thirteen (22% of total) were non-neoplastic (four at the right mammary chain, RMC, and nine at the left mammary chain, LMC), 11 (18% of total) were benign neoplastic (five at RMC and six at LMC), three (5% of total) were in situ malignant neoplastic (two at RMC and one at LMC), and 33 (55% of total) were invasive malignant neoplastic (19 at RMC and 14 at LMC). A summary of the classification results by structural pattern is provided in Table 3.

**Figure 4:**
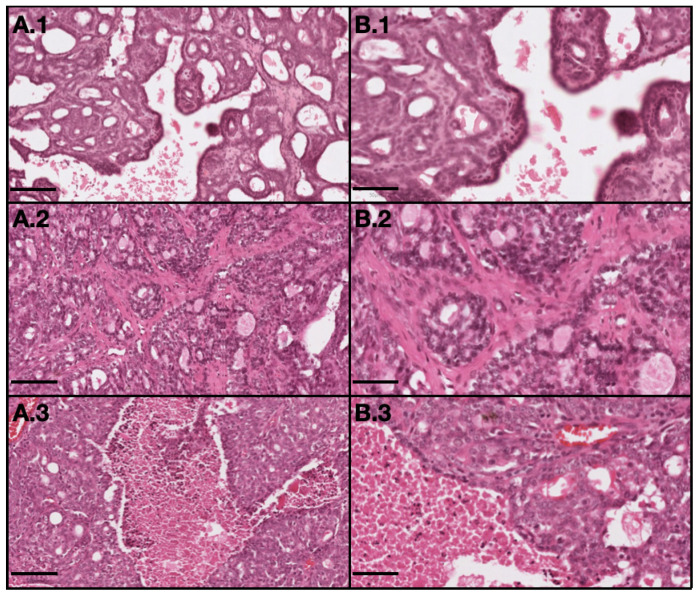
Representative light microscopy images of tumor with different grades: 1–grade I (invasive papillary carcinoma); 2–grade II (invasive tubular carcinoma); and 3–grade III (invasive cribriform carcinoma). (**A**) represents 10× magnification with scale bar = 100 µm and (**B**) represents 20× magnification with scale bar = 50 µm. (H&E).

All benign neoplastic tumors were classified as grade I, as well as all in situ malignant neoplastic tumors. For invasive malignant neoplastic tumors, 20 were grade I, six were grade II and seven were grade III. Among the 33 invasive malignant tumors, 19 were on the RMC (58%) from which 11 (33%) were classified as grade I, five (15%) as grade II tumors and three (9%) as grade III tumors, and 14 (42%) were on the LMC from which 11 (27%) were classified as grade I, five (3%) as grade II tumors and three (12%) as grade III tumors, as described in Table 4. Histological differences between tumors with different grades are showed in Figure 4.

#### 2.1.5. Epigenetic Alterations

##### DNA Methylation

**Figure 5:**
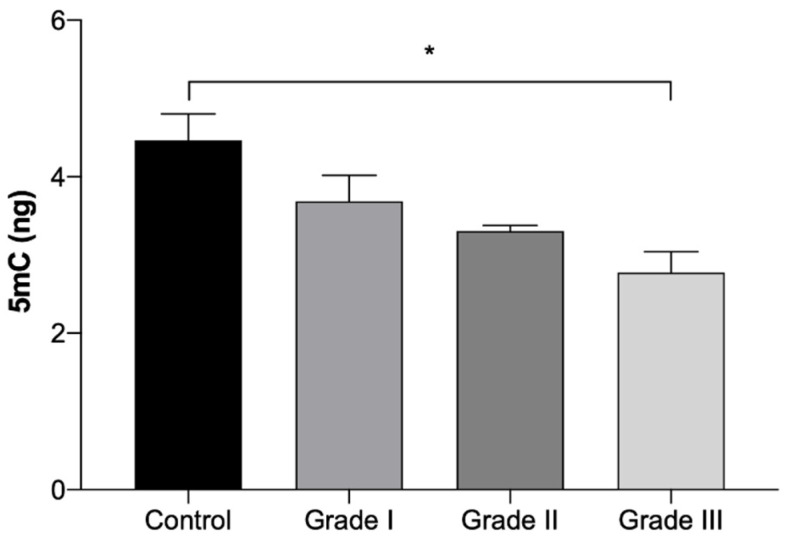
Amount of global 5mC DNA methylation expressed in ng (Mean ± SEM), in normal mammary gland fragments (control) and in invasive malignant tumors fragments, according to the histologically classified tumor grades (Grade I, II and III). Statistically significant differences are identified with * *p* < 0.05.

The results of global 5mC DNA methylation quantification are presented in Table 5 and in Figure 5. The results show a gradual reduction on the 5mC DNA methylation quantification with the worsening of the grade. However, a significant reduction (*p* < 0.05) was only seen in Grade III tumor fragments, which presented a 38% reduction in comparison to the control group. No statistically significant differences were identified between tumors in the right and left mammary chains.

### 2.2. EGF-Conjugated GNPs for Photothermal Treatment

#### 2.2.1. Size, PdI, Maximum Absorbance Peak and Morphology of GNPs

The EGF-conjugated GNPs were prepared and characterized in terms of size, PdI and maximum absorbance peak through all the synthesis steps. The results are summarized in Table 6. Additionally, the size distribution by intensity (%) obtained by DLS are shown in Figure S1. It is noticeable that along the synthesis process, the particles tend to get more stable with smaller sizes and PdI (*p* < 0.05), with the final EGF-conjugated GNPs presenting a main peak size of about 192 nm and a PdI of 0.384. Attending the maximum absorbance peak, upon the addition of the HAOA coating to the Core GNPs without the EGF, a broad band with no defined peak was observed. The final EGF-conjugated GNPs showed a maximum absorbance peak detected by the equipment at 823 nm, which belongs to the NIR range and fulfills the system requirement for the NPs to exhibit a maximum absorbance peak close to the wavelength of the laser source to be used.

**Figure 6:**
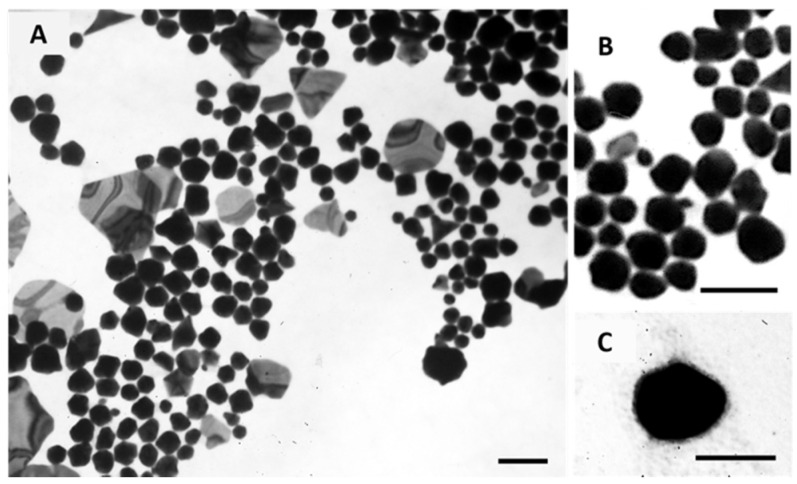
Morphological characterization of Core GNPs (**A** and **B**) and EGF-conjugated GNPs (**C**) by TEM. (**B**) show the spherical-like shape of Core GNPs with more detail. Scale bar = 100 nm.

The morphology of Core GNPs and EGF-conjugated GNPs was observed by transmission electron microscopy (TEM), and the obtained images are shown in Figure 6. These images show a polydisperse population, which is in accordance with the PdI values obtained by DLS. For the Core GNPs, spherical-like GNPs seem to be predominant, although other non-spherical structures are also identified. By its turn, EGF-conjugated GNPs showed more spherical cores, presented as darker inners, with a small coating layer presented as a lighter color cloud-like shell.

#### 2.2.2. In Vitro Photothermal Therapy with Functionalized Gold-Nanoparticles

**Figure 7:**
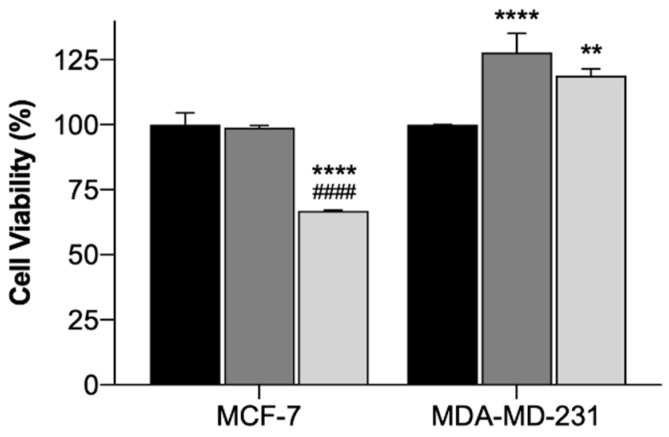
Cell viability (%) of MCF-7 and MDA-MB-231 cells treated with laser irradiation alone (black columns), EGF-conjugated GNPs alone incubated for 4 h (dark grey columns) and EGF-conjugated GNPs incubated for 4 h combined with laser irradiation (light grey columns). The results represent the Mean *±* SD (*n* = 3) and the statistical analysis: ** *p* < 0.01; **** *p* < 0.0001 comparing with the cells only subjected to the laser irradiation and #### *p* < 0.0001 comparing with the cells incubated with EGF-conjugated GNPs alone.

In order to assess the safety and efficacy of using the EGF-conjugated GNPs and the laser irradiation, alone or combined, in breast cancer cell lines, cytotoxicity tests using the MTT assay were conducted. The MTT assay evaluates the mitochondrial activity and it is a standard technique widely used [69]. The results of the incubation of EGF-conjugated GNPs for 4 h and the irradiation with a NIR laser (irradiance of 5.6 ± 0.2 W/cm^2^ during 3 min), alone or combined, onto MCF-7 (Michigan Cancer Foundation – 7) and MDA-MB-231 (M. D. Anderson Cancer Center- MB-231 Cells) cells are presented as cell viability (%) in Figure 7. The use of laser irradiation alone did not affect the cells’ viability of both cell lines, suggesting its safety. EGF-conjugated GNPs alone did not reduce the cells’ viability of either cell lines. However, for MDA-MB-231 cells a slight increase on cell viability was observed. Yet, when extending the EGF-conjugated GNPs’ incubation period up to 24 h, the viability of the cells of either cell lines was not affected (*p* < 0.05), having the MCF-7 and the MDA-MB-231 cells presented 91.6 ± 7.4 (Mean ± SD, *n* = 3) and 99.5 ± 6.5 (Mean ± SD, *n* = 3) cell viability, respectively. Moreover, the application of the EGF-conjugated GNPs combined with laser irradiation led to a reduction of the cells’ viability of MCF-7 cells into 33% (*p* < 0.0001), whereas the MDA-MB-231 showed increased cell viability (*p* < 0.001). Additionally, the efficacy of HAOA-coated GNPs when incubated in both cell lines for 4 h was also tested. The results showed that HAOA-coated GNPs alone did not reduce the cells’ viability (%) of any of the cell lines, neither did the combination of these particles with laser irradiation in MDA-MB-231 cells. In contrast, HAOA-coated GNPs combined with laser irradiation resulted in a reduction of the MCF-7 cells’ viability, which is similar (31%, *n* = 3) to what it was observed for the EGF-conjugated GNPs combined with laser irradiation. This last observation might suggest that the EGF receptor is not the only targeting agent of the GNPs. Like stated in the introduction, CD44 can also have an important role. Further studies should be attempted to elucidate which receptor or receptors are involved in the internalization mechanism of GNPs.

#### 2.2.3. Preliminary Safety Assessment for Potential In Vivo Applications Using Hemolytic Activity Assay

The hemolytic activity of Core GNPs and EGF-conjugated GNPs was determined by using EDTA-preserved peripheral human blood [70] and the results are represented in Table 7. The maximum concentration of particles tested was estimated based on the mass of GNPs to be administered in situ, and considering that a rat has 60 mL/kg of blood volume on average, from which 36–48 vol% correspond to erythrocytes [71]. In this way and considering the worst-case scenario, the maximum concentration tested assumes that 100% of the particles administered in situ reached the blood stream, i.e., none of GNPs remained in the tumor area. The results show no hemolytic effect on either Core GNPs or EGF-conjugated GNPs, which proves that even if the GNPs administered in situ reach the blood stream, they will not cause erythrocytes lysis and are safe to be used.

#### 2.2.4. Preliminary In Vivo Photothermal Therapy with Functionalized Gold-Nanoparticles

**Figure 8:**
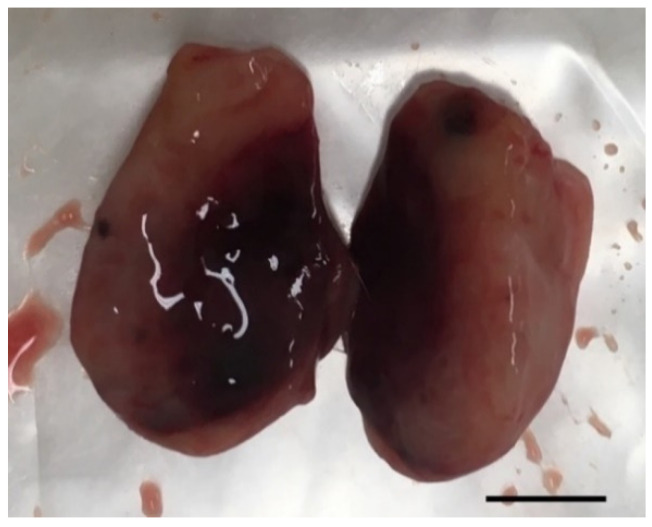
Photography of a representative excised tumor with visible increased hemorrhagic area and where histological analysis revealed a high level of necrosis after treatment with EGF-conjugated GNPs combined with laser irradiation (tumor grade I, scale bar = 0.5 cm).

**Figure 9:**
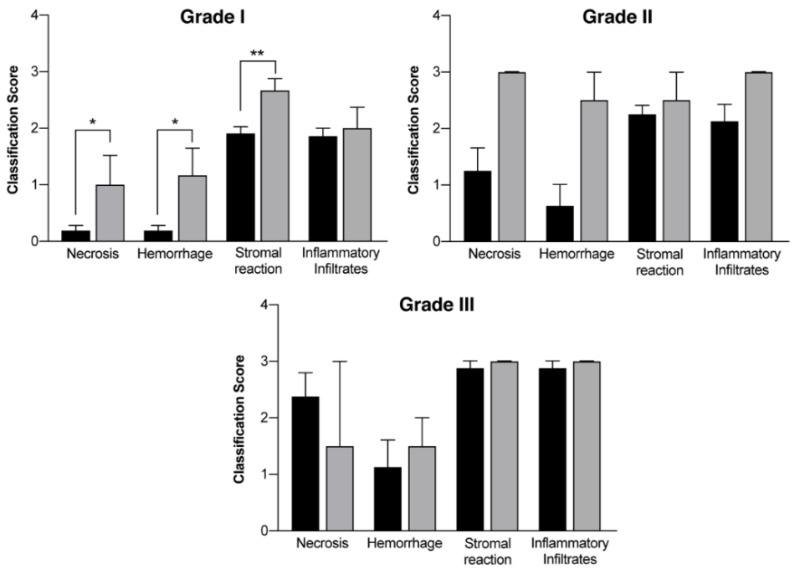
Histological evaluation of mammary tumors according to the different grades (Grade I tumors, Grade II tumors and Grade III tumors) in a group without treatment (DMBA; black columns) and in a group where EGF-conjugated GNPs combined with laser irradiation were used (grey columns). Results are represented as Mean ± SEM and statistically significant differences are identified with * *p* < 0.05 and ** *p* < 0.01.

In vivo mammary tumors’ treatment with EGF-conjugated GNPs associated with laser irradiation showed a macroscopic tumor reduction and an increased hemorrhagic area (Figure 8). When histologically analyzed, that area presented increased necrosis, hemorrhage, stromal reaction and presence of inflammatory infiltrates compared to Grade I and Grade II tumors without treatment, as represented in Table 8 and Figure 9. Non-target organs removed for analysis after necropsy showed no morphological changes.

## 3. Discussion

This work proved DMBA as a very good and selective inductor of breast cancer, with 100% incidence and inducing an average of 4.7 tumors per animal. Moreover, epigenetic findings revealed some hypomethylation in tumors classified with worst prognosis, which unveils the potential of using DNA methylation as a marker to monitor the tumors’ development. Also, the use of GNPs in combination with NIR laser irradiation exhibited encouraging results for the treatment of breast cancer, by showing promising alterations in the treated tumors’ histology.

Animal models represent an important tool for studying various diseases, such as cancer, once they allow to investigate the pathogenesis, progression, and genetic and molecular basis, which enables the development and evaluation of different therapeutic solutions that can improve patients’ quality of life [34,72,73,74]. Although there are no ideal models [75], the choice of an adequate experimental model is crucial, namely the low cost and similarity to what occurs in Humans. In the context of breast cancer models, Sprague-Dawley female rats are the most used strain, although Wistar rats can also be used despite recent studies have showed that the number of mammary tumors in Wistar rats is lower than in Sprague-Dawley rats [5,76].

Sprague-Dawley rats are frequently used in DMBA-induced carcinogenesis studies to generate mammary tumors, having DMBA being reported as an effective inducer of mammary carcinoma after its administration in a single dose [5,6,77]. The time of DMBA administration seems to be crucial since at 21 days of age, the number of terminal end bubs (TEB’s) is maximal and at 30–42 days, hormonal influence of the estrous cycles of puberty stimulates the division of TEB’s and its differentiation into alveolar shoots [78,79]. Moreover, at 55 days, the breast is partially differentiated, and after 55–60 days, chemical carcinogenesis acts on more differentiated alveolar shoots and forms later benign lesions [78,80,81]. Thus, in this work, one single dose of DMBA (65 mg/kg), diluted in virgin olive oil, was dosed by gavage to 50-day-old Sprague-Dawley rats. As a result, high rate of tumors was observed, with 90% of animals in DMBA group developing invasive malignant mammary tumors, with an average of 3.67 tumors per animal. Furthermore, all animals developed mammary tumors in an average of 4.7 tumors per animal. These results also showed a great number of tumors developed in the first four pairs of mammary glands, but no differences in tumors’ development over the right or left mammary chains were seen. The DMBA-induced tumors’ development and distribution do not occur randomly over the six pairs of mammary glands, having been reported that a greater number of tumors develop in the thoracic region rather than in the glands located in abdominal–inguinal region [78], which supports the results herein presented. The experimental model herein developed did not cause metastases, nor any changes in non-target organs, confirmed by the urinalysis and blood analysis. Histopathological analysis highlighted the development of a higher number of grade I invasive malignant tumors than grade II and grade III invasive malignant tumors. Moreover, non-target organs showed no signs of alterations, except for the presence of severe cortical cystic degeneration in adrenal glands, which has also been reported in other works [82,83,84]. Thus, this breast cancer induction model is here presented as an excellent model to study different stages of mammary carcinogenesis without metastasis.

In this work, epigenetic results in mammary tumors revealed a decreasing amount of global 5mC DNA methylation associated with a higher histologically-classified tumor grade, although statistical differences had only been noticed for Grade III tumors. However, the absence of statistical differences on the 5mC DNA methylation over the remaining grade stages comparing to the control group can be a consequence of the smaller sample size of those groups, which means that it is necessary to increase the sampling size to further clarify the relation between grade and the amount of global 5mC DNA methylation. Epigenetic changes, including DNA methylation, for instance, usually occur at the initial stage of tumor progression [85], and have already been associated with cancer development [12,14]. Moreover, it has been hypothesized that they may inappropriately activate or inhibit various signaling pathways. DNA methylation can occur in cytosine carbon 5 (5mC) and it is one of the most studied epigenetic changes, with an overall loss of DNA methylation being a common feature in cancer [86,87]. However, despite the advances in cancer epigenetics over the last years, the determinants of the “epigenetic state” are not fully understood, yet [88].

Overall, this model suggests being very selective and focused on mammary glands, with a high incidence rate of malignant tumors, for the DMBA concentration and other experimental conditions herein used. Moreover, a possible correlation between the increased incidence of breast cancer upon exposure of women at a young age (<17 years) [68] and in adolescence [16,17,18,19,20,21,22,23,24,25,26,27,28] to tobacco has been reported, with the development of breast neoplasms in female rats through the administration of DMBA (compound present in cigarette smoke), at the age of development of TEB’s. Therefore, it seems very resourceful to use this experimental model, since some characteristics are common in humans.

Concerning breast cancer therapy, and as previously stated, there are several treatment options such as surgery, radiation and/or chemotherapy. Immunotherapy and some nanoparticle-based therapies have also been approved [89]. Besides not being possible to perform in all the patients, the surgical removal of the tumor may not be the best treatment option in eligible patients since it may lead to breast deformities with a great impact on the patients’ life and self-esteem [90]. Radiation and chemotherapy are commonly used alone or in combined treatments. However, the mechanism of its action is not specific towards cancer cells, and it usually damages healthy tissues. Despite the promising developments, chemotherapy agents are still given systemically, increasing the occurrence of side effects, even though the doses are optimized for a more selective action [91]. For this purpose, photothermal therapy (PTT) is emerging as clinically viable and of great interest in superficial cancers treatment like breast cancer. The photothermal therapeutic effect relies on the use of NIR radiation and on the administration of nanoparticles, such as GNPs, into the tumors.

The size-dependent toxicity and cellular uptake of GNPs is still debatable, as it is known that different sizes and shapes are translated in different physicochemical properties and, consequently, different biological effects can occur. In this work, it is intended to have a localized effect without affecting non-target organs, which implies that the particles must have a size range that hinders the possibility of entering the blood flow and accumulate in vital metabolic organs, as well as delaying their removal by immune system elements. Some groups have already reported that smaller nanoparticles (<20 nm) are able to pass the blood-brain barrier [92,93] and the placental barrier [94], and can permeate the skin and intestine better than bigger particles (≈ ≥ 200 nm) [92,95]. Thus, for this work, particles of about 200 nm seem more suitable to be localized in the target area, although the GNPs biodistribution upon their administration must be assessed since some reported the preferential accumulation of GNPs in the liver upon their intravenous administration [96]. The EGF-conjugated GNPs were fully prepared and characterized regarding their size, PdI and maximum absorbance peak over the diverse synthesis steps. Upon the progression over the synthesis steps it was noticed that the particles got more stable, showing both size and PdI reduction. The same tendency was observed by TEM analysis, which showed not only the size and PdI reduction over the production phases, but also the polydispersity of the particles’ population, specially of the Core GNPs, through the observation of different GNPs’ shapes. Those results are in accordance with what was previously published by our group [55]. The final EGF-conjugated GNPs obtained presented an average diameter of about 192 nm, which is slightly different from the particles’ sizes of about 220 nm that were previously reported by our group [55,58]. In the same work it was also reported a size reduction of about 27% upon the addition of the EGF in comparison to the HAOA-coated GNPs, whereas herein a 43% size reduction was seen. This decrease might be attributed to some rearrangement of the GNPs structure. Furthermore, our group previously reported a maximum absorbance peak of around 655 nm for the EGF-conjugated AuNPs in contrast with a maximum absorbance peak of around 800 nm for the Core GNPs, whereas herein the same particles showed a maximum absorbance peak of 823 nm and 899 nm, in the respective order. Despite the particles’ features differences highlighted between the results here presented and the previously reported by our group, it must be kept in mind that small changes were made in the protocol, so a direct comparison between the results cannot be done. In summary, herein it is reported the synthesis of bigger particles but with maximum absorbance peak in the NIR region and closer to the wavelength of the laser used in the combined system proposed.

The in vitro tests results suggest the safety and inefficacy of the use of laser irradiation alone to kill cancer cells. These results are in agreement with what other groups published for the use of laser irradiation within the same wavelength range and with higher irradiances [97]. It was also showed that the EGF-conjugated GNPs alone, incubated for 4 h, did not reduce the cells’ viability (%), which also confirms their safety and inefficacy as anti-cancer therapy. Furthermore, these particles alone seemed to increase the cell’ viability of MDA-MB-231 cells, which can raise suspicions that the formulation might promote the growth of these cells. However, when the same particles were incubated for longer periods of time (24 h), no alteration of the cells’ viability was seen, which devalued the previous suspicions regarding the promotion of MDA-MB-231 cellular growth. Nevertheless, the increase observed in the cell’ viability of the MDA-MB-231 cells incubated with the NPs alone is still not understood by the authors, so further tests must be conducted. The combination of EGF-conjugated GNPs and HAOA-coated GNPs with laser irradiation led to a reduction of about 33% (*p* < 0.0001) and 31% of MCF-7 cells’ viability, respectively, when comparing to non-treated cells. The slight increase of the MDA-MB-231 cells’ viability after the use of the combined treatment reflects the same behavior of using the NPs alone, although the increase resultant from the combined treatment has been slightly smaller than the one observed for NPs alone. Nevertheless, the combination of the HAOA-coated and EGF-conjugated GNPs with laser irradiation showed better results than NPs alone in both cell lines, which was translated in a reduction of the cell’ viability. These results raised suspicion on the value of using EGF in the system for the particular cell lines herein used, since the coated GNPs showed similar results to the ones from EGF-conjugated GNPs. However, it must be kept in mind that the cells receptors expression of these cells was not assessed. And even though, according to the literature both cell lines overexpress EGFR [61,98], it is also known that upon subsequent cell replications the probability of occurring genomic changes and mutation increases [29]. When comparing the results from the two cell-lines it is clear that the combined treatment had a far better efficacy over MCF-7 cells than MDA-MB-231 cells, thus requiring complementary further tests to clarify the mechanisms underlying these findings. According to the literature, it is reasonable to hypothesize that the different response to treatment of the two cell lines might be a consequence of the different expression of EGFR and the different response to EGFR-targeted treatments [61], the different expression of CD44 [59], as well as the different metabolic mechanisms of the two cell lines [98]. Moreover, it might also be related with the different tumor types that each cell line represents and their hormonal receptors expression. MCF-7 represents a ER and PR positive and HER2 negative breast cancer [31,99], whereas MDA-MB-231 represents a triple-negative breast cancer [31,100], which in the literature is associated to poor prognosis and fewer and less efficient treatments available [101]. Furthermore, the hemolytic activity results proved that for the maximum concentration of GNPs tested, corresponding to the hypothesis that 100% of the GNPs administered in vivo would be able to enter the blood stream, the GNPs will not cause the lysis of the erythrocytes, which supports their safety for in vivo applications.

The receptors expressed by the tumors developed in vivo were not assessed, so EGF-conjugated GNPs were used as a potential more targeted tool for tumor cells in comparison to healthy cells, based on the increased expression of EGFR in tumor cells reported in literature [61]. Macroscopically, this study suggests a tumor volume reduction immediately after the treatment with EGF-conjugated GNPs combined with laser irradiation, without showing any skin burn. After excision, it was observed a tumor area completely isolated and with hemorrhagic aspect (Figure 8). The histopathological analysis showed an increase of necrosis, hemorrhage, stromal reaction and presence of inflammatory infiltrates in non-treated higher-grade tumors (Grade II and III), generally associated with a more aggressive growth. Additionally, it revealed that upon the treatment of Grade I tumors with the GNPs combined with laser irradiation, the necrosis, hemorrhage and stromal reaction increased comparing to non-treated tumors from the same grade. These findings, associated to the macroscopic tumor volume reduction observed, may suggest that necrosis might be one of the mechanisms behind the tumor reduction. However, the precise mechanism of cell’ death is still not completely understood.

## 4. Materials and Methods

### 4.1. In Vivo Studies

Sprague-Dawley female rats with 6 weeks old supplied from Charles River (Barcelona, Spain) were housed in polypropylene cages at ambient temperature (20–24 °C), relative humidity (55 ± 5%), 12 h light/dark cycle and given standard diet and water *ad libitum*. All animal experiments were conducted according to the animal welfare organization of the Faculty of Pharmacy (ORBEA), University Coimbra, and approved by the competent national authority Direção-Geral de Alimentação e Veterinaria (DGAV) (Title: Oncotherapy in an experimental model of breast cancer, Ref. DGAV/01/18) and in accordance with the EU Directive (2010/63/EU), the Portuguese laws (Law 113/2013, 2880/2015, 260/2016 and 1/2019) and all relevant legislation. This study was divided into two parts: Experimental model characterization and EGF-conjugated GNPs combined with laser irradiation for photothermal therapy.

#### Development and Characterization of an Experimental Model

Sprague-Dawley female rats (*n* = 20) were randomly divided in two groups: Animals with no manipulation (Control group, *n* = 10); and animals dosed with DMBA (DMBA group, *n* = 10). All animals were observed and weighted weekly.

At 50 days of age, the animals from the test group were orally administered with 65 mg/kg of DMBA dissolved in virgin olive oil. Fifteen weeks after carcinogenic induction, tumors started to be detectable by mammary palpation. Dimensions of the mammary tumors and signs of lameness, paralysis or weakness were controlled during every week of the experimental protocol. At 27 weeks, after chemically-induced breast cancer, blood samples were collected from each animal and a full necropsy was performed. All tumors were excised, measured, weighed, photographed and frozen at −80 °C in 10% formalin.

Mammary glands of each mammary chain were numbered by the nipple from one to six in the cranio-caudal direction, and the mammary chains were divided into right mammary chain (RMC) and left mammary chain (LMC).

1. Urinalysis

Once a month, each animal was placed in a metabolic cage for 24 h. During the experimental protocol, six urine samples were collected from each animal. After collection, samples were stored at −20 °C until required urine analysis. At the time of analysis, all urine samples were defrosted and manually agitated before being transferred to 10 mL tubes for posterior analysis. To perform the physicochemical tests, reactive strips (Combiscreen 10 sl, Analyticon Biotechnologies AG, Lichtenfels, Germany) were put in contact with the urine and read on the automatic device (Combiscan 100 from Analyticon Biotechnologies AG, Lichtenfels, Germany) using refractometry technique. After, the urine samples were centrifuged at 1250× *g* for 10 min (Kubota 5900, Tokyo, Japan), in order to obtain and separate the supernatant. Approximately 500 µL of supernatant were transferred to secondary tubes to perform the urea and creatinine quantification, using the biochemistry auto-analyzer (Olympus AU400 from Beckman-Coulter, California, USA). Another part of the supernatant (200 µL) was diluted ten times with a specific reagent to perform the urinary ionogram (Na^+^, K^+^, Cl^−^), using potentiometry as method for determination on an automatic device (Spotlyte from A. Menarini, Florence, Italy).

2. Blood Samples Analysis

- Hematological Parameters

One sample per animal was obtained at the time of sacrifice. The blood samples were collected into tubes containing EDTA K_3_ and kept under mechanical agitation until analysis. The samples were then analyzed in an automatic cell counter (HMX from Beckman-Coulter, California, USA) to quantify erythrocytes, hemoglobin, hematocrit (HCT), mean corpuscular volume (MCV), mean corpuscular hemoglobin (MCH), mean corpuscular hemoglobin concentration (MCHC), leucocytes, neutrophils, lymphocytes, monocytes, eosinophils and platelets.

- Biochemical Parameters

Samples were centrifuged at 1500× *g* for 20 min (Kubota 5900, Kubota Co.,Tokyo, Japan), allowing the separation of the serum into primary tubes. Then, a part of the serum (approximately 500 µL) was put in a secondary tube. Glucose, urea, creatinine, blood urea nitrogen (BUN), aspartate transaminase (AST), alanine transaminase (ALT), alkaline phosphatase (ALP) and total calcium were quantified using the biochemistry auto-analyzer and colorimetry, enzymatic colorimetry and enzymatic kinetics methodologies.

3. Histopathological Assessment

All collected samples were fixed in 10% buffered formalin and then routinely processed for paraffin embedding. After embedding, 3 µm sections were prepared for conventional heamatoxylin-eosin staining (H&E). Histopathological assessment was performed using a conventional light microscope (Olympus CX21) and images were acquired using a NanoZoomer-SQ Digital slide scanner (Hamamatsu Photonics).

Lesions were graded as non-neoplastic, benign neoplastic, in situ malignant neoplastic and invasive malignant neoplastic, and categorized based on their predominant growth pattern, according to Russo 2015 [102]. The total number of patterns was recorded. In cases that presented malignant and benign lesions, the histological type given was the predominant on the malignant lesion. In the benign lesions, all patterns were considered.

Grading of malignant lesions was also performed applying the Nottingham Grading System (NGS) (suggested by the World Health Organization to classify woman breast neoplasia) [103]. This system is based on evaluating and scoring three distinct morphological features: degree of tubular/glandular formation, nuclear pleomorphism and mitotic index [77,103,104]. For tubular/glandular formation, a score of 1 was given when this pattern was present in more than 75% of the lesion; a score of 2 when this percentage was between 10–75% and a score of 3 when it was below 10%. Nuclear pleomorphism was classified as: 1 when the cells presented a regular form and a small size; 2 when moderate size and shape variation was observed; and 3 when severe shape and size variation between cells was seen. The mitotic count was performed on 10 consecutive high-power fields (Obj 40×, FN 22), on the tumors’ peripheral area. A score of 1 was given when total mitotic count was below 11, a score of 2 when it was between 12–22 and, finally, a score of 3 when mitotic total was over 23. After scoring these features, a grade was given to the tumor based on the sum of the three results. A tumor that scored 3–5 points was classified as grade I, grade II was given to tumors with scores between 6–7 and grade III for those scoring 8–9 points.

Furthermore, non-target organs were collected to assess systemic DMBA toxicity and the appearance of potential metastases.

4. DNA Extraction

Fragments of invasive malignant neoplastic tumors (*n* = 28), frozen at −80 °C and histologically classified as grade I (*n* = 16), grade II (*n* = 6) and grade III (*n* = 6), were defrosted immediately before use. DNA was extracted from 25-30 mg of mammary gland tissue samples and isolated using the DNeasy Blood and tissue isolation kit (Qiagen GmbH, Germany) following the manufacturer’s protocol. The purity and concentration of DNA were measured by NanoDrop^TM^ 1000 Spectrophotometer (Thermo Scientific, USA) with an absorbance at 280-260 nm and 260-230 nm, respectively. At 230–260 nm, the absorbance measured was 1.98 ± 0.06 with CI95% = [1.85; 2.11] and at 260–280 nm it was 1.84 ± 0.02 with CI 95% = [1.81; 1.87]. This translates a high degree of purity, without contamination.

5. DNA Methylation

The ELISA-based “Methylated DNA Quantification Kit (Colorimetric)” (Abcam ab117128, Cambridge, UK) was used to quantify global 5mC DNA methylation content in control mammary tissue and DMBA invasive malignant breast tumors. The assay was performed in duplicates according to the manual, and the absorbance was read at 450 nm. The input DNA was diluted in TE buffer (Tris-EDTA buffer) to an optimum 100 ng per reaction.

The commercial negative control was used to subtract the value of relative absorbance units for all measurements for background absorbance correction. For quantification, a calibration curve for each experimental condition (0.5, 1.0, 2.0, 5.0 and 10.0 ng/µL) was built. With this curve, the levels of 5mC using the Equation (1) were calculated, where 2 is a factor to normalize 5mC in the positive control to 100%, as the positive control contains only 50% of 5 mC, sample OD is the optical density of samples and Negative Control OD is the optical density of the negative control sample.

(1) 5 mC (ng) = (Sample OD−Negative Control OD)÷(slope × 2)

### 4.2. EGF-Conjugated GNPs Preparation and Characterization

#### 4.2.1. EGF-Conjugated GNPs Preparation

The EGF-conjugated GNPs were prepared upon adaptation of the method previously described by our group [55,58,105]. It consists of binding EGF onto the surface of core GNPs coated with a mixture of hyaluronic acid (HA) and oleic acid (OA). Briefly, the core (Core GNPs) was firstly prepared based on a mixture (1:4, *v/v*) of reducing agents (Rosmarinic Acid (3.5 mM), L-ascorbic acid (2 mM), silver nitrate (AgNO_3_, 1 mM)) with gold (III) chloride trihydrate solution (HAuCl_4_·3H_2_O, 1 mM) at room temperature (RT), under magnetic stirring (800 rpm) (Heidolph MR3001, Heidolph Instruments, Schwabach, Germany) for 15 min. The HAOA coating was prepared upon the dissolution of 5 mg of HA sodium salt from *Streptococcus equi* and 20 µL of OA in 5 mL of Milli-Q water at 60 °C under magnetic stirring (400 rpm) overnight. Then, the HAOA coating was added to the Core GNPs on a 1:1 (*v*/*v*) ratio. It was immediately followed by the addition of an EGF solution in PBS (1 mg/mL) on a 1:1 (*v*/*v*) ratio, which was allowed to react under magnetic stirring (800 rpm) at RT for 30 min. Afterwards, the particles were stored for 24 h at 2 °C protected from the light, and then centrifuged at 7200× *g* during 15 min to remove the unbound EGF. All the reagents used in the particles’ preparation were purchased from Sigma-Aldrich (St. Louis, MO, USA), except in the case of the EGF recombinant human protein, which was purchased from ThermoFisher Scientific (Walthman, MA, USA).

#### 4.2.2. Characterization of the GNPs

The GNPs were characterized over the consecutive synthesis stages (Core GNPs, HAOA-coated GNPs and EGF-conjugated GNPs) in terms of their mean particle size and their polydispersity index (PdI) using dynamic light scattering (Zetasizer Nano S, Zen 1600, Malvern Instruments, Malvern, UK) at a constant temperature of 25 °C and a 173° scattering angle. This characterization was made in diluted samples (with Milli-Q water, 1:6). For every sample, 3 series of 11 measurements were made. The particles’ maximum absorbance peak was obtained by single measurements using spectrophotometry (Shimadzu UV-160A UV-visible recording spectrophotometer, Shimadzu Europe GmbH, Duisburg, Germany).

Moreover, the particles’ morphology was analyzed through Transmission Electron Microscopy (TEM). Ten microliter droplets of the GNPs aqueous suspensions were applied on 200-mesh copper grids coated with formvar and carbon. The particles were allowed to attach to the formvar/carbon film for a few minutes, and later, the excess of the samples was removed with a piece of filter paper. Next, the material was negatively stained with 1.0% uranyl acetate for some minutes and left to dry at room temperature. Observations were carried out on a JEOL 1200 EX transmission electron microscope (JEOL Ltd., Tokyo, Japan) at 80 kV and images of diverse grid fields were recorded digitally.

#### 4.2.3. In Vitro Photothermal Therapy with Functionalized Gold-Nanoparticles

##### Cell Culture and Incubation with the EGF-Conjugated GNPs

To conduct the in vitro safety and efficacy studies, human breast cancer MCF-7 (ATCC^®^ (American Type Culture Collection) HTB-22^TM^, ATCC, Manassas, VA, USA) and MDA-MB-231 (ATCC^®^ HTB-26^TM^, ATCC, Manassas, VA, USA) cell lines were selected. MCF-7 cell line represents an ER and PR positive and HER2 negative cancer, whereas MDA-MB-231 cell line represents a triple-negative cancer. Both cell lines were cultured in Dulbecco’s Modified Eagle’s medium (DMEM) with high-glucose (4500 mg/L) enriched with 10% Fetal Bovine Serum, 100 IU/mL of Penicillin and 100 µg/mL of Streptomycin (Invitrogen, Carlsbad, CA, USA) (henceforward, complete medium), in an incubator at 37 °C and 5% CO_2_ atmosphere. Then, on the day before the GNPs’ incubation day, the cells were seeded in 96-well plates at a concentration of 5 × 10^4^ cells/mL. In the case of the plates with the cells being posteriorly subjected to laser irradiation, alone or in combination with EGF-conjugated GNPs, one empty well was kept in all the directions surrounding the wells containing the cells to be irradiated, to ensure that those cells would not receive any scattered or reflected light by the neighbor wells. The cells were incubated with EGF-conjugated GNPs in complete medium at a concentration of 50 µM of gold for 4 h, a period of time in which the internalization of the particles was already reported by our group [1]. After the 4 h, the medium was removed to retrieve the NPs not bound/internalized and fresh complete medium was added. Note that even the medium from the cells not incubated with NPs was removed to ensure that all the test groups were under the same conditions.

##### Laser Irradiation Procedure

The cells were irradiated with a wavelength of 811 nm JDSU L4-2495-003 Diode Laser (JDSU, San Jose, CA, USA) coupled to a LaserPak laser diode driver ARO-485-08-05 (Arroyo Instruments, LCC, San Luis Obispo, CA, USA). The laser beam was collimated so, regardless the distance between the laser output and the cells, the beam size was constant (5.7 ± 0.1 mm) and centered in the middle of each well. This, in combination with the use of the same intensity in all the irradiations, made it possible to subject all the cells to the same irradiance (5.6 ± 0.2 W/cm^2^). Moreover, all cells were irradiated uninterruptedly during 3 min, which corresponded to an energy density of 10.2 ± 0.4 J/mm^2^.

##### MTT Assay

After the laser irradiation procedure, or simply 4 h or 24 h after the incubation of the particles in the case of the cells not receiving laser irradiation, the complete medium was removed, and the cells were washed twice with phosphate buffered saline (PBS). Then, an MTT solution at 0.5 mg/mL in incomplete medium was added and the cells were, again, incubated for 4 h. Later, in order to dissolve the formazan crystals produced by the cells upon the reduction of the MTT, 100 µL of Dimethyl Sulfoxide (DMSO) was added. The absorbance at 570 nm was measured using a BioTek ELx800 Absorbance Microplate Reader (BioTek Instruments, Inc., Winooski, VT, USA). Cells’ viability in percentage was determined according to the equation,

(2) $$\mathrm{Cell}\;\mathrm{Viability}\;\left(\%\right)\;=\frac{\mathrm{ODt}}{\mathrm{ODc}}\times 100$$

where ODt is the optical density of cells incubated with the tested formulations and ODc is the optical density of the control cells, corresponding to 100% cell viability.

#### 4.2.4. Preliminary Safety Assessment for Future In Vivo Applications Using Hemolytic Activity Assay

The hemolytic activity of GNPs in different forms was evaluated using EDTA-preserved peripheral human blood [70] collected from voluntary donors and used in the same day of the experiments. The blood sample was centrifuged at 1000× *g* for 10 min to allow the removal of the serum. This was followed by three washes of the erythrocyte suspension in PBS, using 1000 × *g* for 10 min (Beckman GPR Centrifuge, Beckman Coulter, Inc., Brea, CA, USA). The GNPs re-suspended in PBS were distributed in 96-well plates (100 µL per well) in concentrations ranging from 1.4 to 0.0006 mg/mL. Furthermore, 100 µL of distilled water and PBS were transferred into six wells each, to work as positive (100% hemolysis) and negative (0% hemolysis) controls, respectively. Then, 100 µL of erythrocytes suspension were added to all the wells with samples, and the plates were incubated at 37 °C for 1 h. Next, the plates were centrifuged at 1000× *g* for 10 min, 50 µL of the supernatant from each well were carefully collected and the absorbance was measured at 570 nm with a reference filter at 630 nm using a BioTek ELx800 Absorbance Microplate Reader (BioTek Instruments, Inc., Winooski, VT, USA). The percentage of hemolysis was calculated for each sample in accordance with the Equation (3):

(3) $$\mathrm{Hemolysis}\;\left(\%\right)\;=\frac{\mathrm{AbsS}-\mathrm{AbsN}}{\mathrm{AbsP}-\mathrm{AbsN}}\times 100$$

where AbsS is the absorbance of the sample, AbsN is the average absorbance of the negative control and AbsP is the average absorbance of the positive control.

#### 4.2.5. In Vivo Photothermal Therapy with Functionalized Gold-Nanoparticles

Sprague-Dawley rats were used for treatment with a photothermal approach with functionalized gold-nanoparticles combined with laser irradiation. After DMBA administration and tumor development, tumors were injected with a solution of EGF-conjugated GNPs suspended in PBS. A total of 10 tumors received approximately 7 mg of the solution containing the NPs. After the injection, rats remained in their cages during 4 h to allow NP’s spreading through the tumor and their internalization by the tumor’ cells. Then, laser irradiation was applied to the center of the previously injected tumors using an 808-nm wavelength RLTMDL-808 diode laser coupled to an optical fiber with 0.22 numerical aperture (Roithener LaserTechnik GmbH, Vienna, Austria). The irradiation was performed with the laser placed 7 cm away from the tumor, having an irradiance of 1.02 ± 0.08 W/cm^2^.The irradiation was kept for 120 s, which corresponds to an energy density of 1.2 ± 0.1 J/mm^2^. Twenty-four hours after treatment, blood samples were collected from each animal and a full necropsy was performed. All tumors were excised, measured, weighed and photographed. The tumors were measured using a caliper, and their volumes were calculated based on the Equation (4):

(4) $$\mathrm{Tumor}\;\mathrm{volume}\;=\;\frac{W^{2}\times L}{2}$$

where W is the tumor width, corresponding to the smallest diameter, and L is the tumor length, corresponding to the bigger diameter and perpendicular to the width [106]. Histopathological assessment of each tumor was performed, and the tumors were classified with a score between 0 and 3, to access relevant morphological characteristics such as necrosis, hemorrhage, stromal reaction and the presence of inflammatory infiltrates (Table 9). Non-target organs were also analyzed. The scores of the tumors treated with EGF-conjugated GNPs combined with laser irradiation were compared with the DMBA tumors scores. This last assay was done using a very small number of animals because it represents a proof-of-concept of these technology and the 3R’s principles were always respected.

### 4.3. Statistical Analysis

For the characterization of the experimental model through urinalysis and histopathological assessment, descriptive statistics were made. In blood samples analysis, the significance of differences was assessed using the Mann-Whitney test, to compare all parameters between the control and the test group. In the quantification of methylated DNA, the significance of differences was assessed using one-way ANOVA followed by Tukey’s multiple comparisons test. To compare the particles’ features over different synthesis stages of the EGF-conjugated GNPs, statistical analysis was done using the one-way ANOVA followed by Tukey’s multiple comparisons test. For the in vitro safety and efficacy assessment of the use of EGF-conjugated GNPs and laser irradiation, alone or combined, the statistical differences were analyzed using the two-way ANOVA followed by Tukey’s multiple comparisons test. To evaluate the histological alterations of GNPs, Mann-Whitney test was applied, comparing tumors between DMBA group and EGF-conjugated GNPs group. For normality test, the Shapiro-Wilk test was made. All results were expressed as mean ± standard error of the mean (SEM), except for the particles characterization and cell viability (%) data which are represented as mean ± standard deviation (SD). The differences were considered significant when *p*-value < 0.05. All statistical analysis was performed using GraphPad Prism 8^®^ (San Diego, CA, USA).

## 5. Conclusions

This work showed that chemically-induction of breast cancer using DMBA is an important and helpful experimental model to breast cancer research. This experimental model was easy to perform, and it was specific for mammary glands. The exact protocol is now well-defined and fully characterized in terms of dose, route and age of incidence. The number of induced tumors was high: 100% of the animals had tumors and the ratio was 4.7 tumors per animal. This work also confirms that DNA methylation analysis seems to be a potential marker in monitoring the development of tumors. Moreover, it was concluded that PTT using laser source and EGF-conjugated GNPs alone were both safe. However, when combined, this approach using functionalized GNPs and laser irradiation resulted in a decrease of cell’ viability in MCF-7 cells and, in vivo, in different levels of necrosis, grade-dependent.

Thus, the full characterization of this experimental model can be considered as an exciting step forward in breast cancer research, closely mimicking a real human breast tumor and thus an important key tool to test the previous strategy or many other potential therapeutic strategies. Given these preliminary results, PTT and GNPs also demonstrated to have a great potential to expand breast cancer treatment options. Further research will assess if this strategy can be applied as an adjuvant technique to surgical intervention, improving, at least, esthetic outcomes, or applied alone when other therapies are not viable, safe, or acceptable.

## Figures and Tables

**Table 1:** Summary of the urine samples analysis of the several measured urine parameters and their units of quantification (*n* = 10 each group per month over 6 months).

Parameter	Units	Control Group		DMBA Group	
		Absolute N. of Samples	% of Samples	Absolute N. of Samples	% of Samples
**Bilirubin**	Negative 1 mg/dL 2 mg/dL 4 mg/dL	59 1 0 0	98.33 1.67 0.00 0.00	59 1 0 0	98.33 1.67 0.00 0.00
**Urobilinogen**	Normal 2 mg/dL 4 mg/dL 8 mg/dL 12 mg/dL	60 0 0 0 0	100.00 0.00 0.00 0.00 0.00	60 0 0 0 0	100.00 0.00 0.00 0.00 0.00
**Ketone**	Negative 10 mg/dL 25 mg/dL 100 mg/dL 300 mg/dL	60 0 0 0 0	100.00 0.00 0.00 0.00 0.00	60 0 0 0 0	100.00 0.00 0.00 0.00 0.00
**Ascorbic acid**	Negative 20 mg/dL 40 mg/dL	3 57 0	5.00 95.00 0.00	3 57 0	5.00 95.00 0.00
**Glucose**	Normal 50 mg/dL 100 mg/dL 250 mg/dL 500 mg/dL 1000 mg/dL	60 0 0 0 0 0	100.00 0.00 0.00 0.00 0.00 0.00	60 0 0 0 0 0	100.00 0.00 0.00 0.00 0.00 0.00
**Protein**	Negative 30 mg/dL 100 mg/dL 500 mg/dL	38 14 4 4	63.33 23.33 6.67 6.67	47 6 2 5	78.33 10.00 3.33 8.33
**Erythrocytes**	Negative 5-10 Ery/µL 50 Ery/µL 300 Ery/µL	47 11 2 0	78.33 18.33 3.33 0.00	44 16 0 0	73.33 26.67 0.00 0.00
**pH**	5 6 6.5 7 7.5 8 9	41 15 0 2 0 2 0	68.33 25.00 0.00 3.33 0.00 3.33 0.00	29 27 0 2 0 0 2	48.33 45.00 0.00 3.33 0.00 0.00 3.33
**Nitrite**	Negative Positive	59 1	98.33 1.67	59 1	98.33 1.67
**Leukocytes**	Negative 25 Leu/µL 75 Leu/µL 500 Leu/µL	60 0 0 0	100.00 0.00 0.00 0.00	60 0 0 0	100.00 0.00 0.00 0.00

Classification based on the Combiscan 100 from Analyticon Biotechnologies AG parameters.

**Table 2:** Summary of the blood samples analysis, expressed as Mean ± SEM and CI95%, of the several measured blood parameters and units of quantification (*n* = 10 each group).

	Control		DMBA		Unit
	Mean ± SEM	CI 95%	Mean ± SEM	CI 95%	
**Erythrocytes**	7.4 ± 0.1	[7.1;7.7]	7.6 ± 0.2	[7.2; 8.0]	10^12^/L
**Hemoglobin**	137.6 ± 2.5	[131.7; 143.6]	141.8 ± 3.6	[133.3; 150.2]	g/L
**HCT ^1^**	0.41 ± 0.01	[0.40; 0.43]	0.43 ± 0.01	[0.40; 0.46]	l/L
**MCV ^2^**	55.0 ± 0.5	[53.8; 56.1]	56.7 ± 0.9	[54.7; 58.8]	fL
**MCH ^3^**	18.5 ± 0.2	[18.1; 18.9]	18.7 ± 0.3	[17.9; 19.5]	pg
**MCHC ^4^**	336.9 ± 1.1	[334.2; 339.5]	329.1 ± 3.4	[321.1; 337.1]	g/L
**Leucocytes**	5.0 ± 0.7	[3.3; 6.6]	6.2 ± 1.4	[2.9; 9.6]	10^9^/L
**Neutrophils**	12.6 ± 2.4	[6.8; 18.4]	22.1 ± 6.7	[6.4; 37.9]	%
**Lymphocytes**	80.5 ± 2.4	[74.8; 86.2]	72.6 ± 7.1	[55.9; 89.3]	%
**Monocytes**	5.2 ± 0.6	[3.7; 6.8]	4.0 ± 0.9	[1.9; 6.0]	%
**Eosinophils**	1.6 ± 0.5	[0.4; 2.9]	1.2 ± 0.4	[0.3; 2.2]	%
**Platelets**	737.4 ± 27.7	[671.9; 802.9]	629.6 ± 60.1	[487.6; 771.6]	10^9^/L
**Glucose**	259.3 ± 29.4	[189.8; 328.7]	240.8 ± 22.8	[186.7; 294.8]	mg/dL
**Urea**	40.0 ± 1.8	[35.8; 44.2]	35.6 ± 4.6	[24.6; 46.6]	mg/dL
**Creatinine**	0.57 ± 0.03	[0.50; 0.65]	0.52 ± 0.02	[0.47; 0.57]	mg/dL
**BUN ^5^**	18.7 ± 0.8	[16.7; 20.6]	16.7 ± 2.2	[11.5; 21.8]	mg/dL
**AST ^6^**	192.9 ± 39.4	[99.7; 286.1]	164.1 ± 20.5	[115.6; 212.7]	U/L
**ALT ^7^**	47.6 ± 4.9	[36.0; 59.3]	40.6 ± 7.5	[22.9; 58.3]	U/L
**ALP ^8^**	72.2 ± 5.5	[59.3; 85.2]	71.2 ± 11.5	[44.0; 98.4]	U/L
**Calcium**	11.2 ± 0.4	[10.3; 12.1]	12.1 ± 0.8	[10.1; 14.1]	mg/dL

**^1^** Hematocrit; **^2^** Mean corpuscular volume; **^3^** Mean corpuscular hemoglobin; **^4^** Mean corpuscular hemoglobin concentration; **^5^** Blood urea nitrogen; **^6^** Aspartate transaminase; **^7^** Alanine transaminase; and **^8^** Alkaline phosphatase.

**Table 3:** Classification of tumors by structural pattern.

	N. Tumors	% Tumors	RMC ^1^	LMC ^2^
**Non-neoplastic**	13	22	4	9
**Benign neoplastic**	11	18	5	6
**In situ malignant neoplastic**	3	5	2	1
**Invasive malignant neoplastic**	33	55	19	14
**Total**	60	100	30	30

^1^ Right mammary chain; ^2^ Left mammary chain.

**Table 4:** Classification of invasive malignant tumors distributed over the six pairs of mammary glands based on the Nottingham grading system (NGS).

	Absolute N. of Tumors	% Tumors	Grade I (Absolute N.)	Grade II (Absolute N.)	Grade III (Absolute N.)
RMC ^1^	19	58	11	5	3
LMC ^2^	14	42	9	1	4
Total	33	100	20	6	7
1st pair	7	21	4	0	3
2nd pair	6	18	3	1	2
3rd pair	8	24	6	1	1
4th pair	9	27	6	2	1
5th pair	1	3	0	1	0
6th pair	2	6	1	1	0

^1^ Right mammary chain; ^2^ Left mammary chain.

**Table 5:** Quantification of global 5mC DNA methylation expressed in ng (Mean ± SEM), in normal mammary gland fragments (control) and in invasive malignant tumors fragments, according to the histologically-classified tumor grades (Grade I, II and III), divided by mammary chains.

		5 mC	
		Mean ± SEM	CI 95%
**Control (*n* = 12)**	LMC ^1^	4.782 ± 0.610	[3.088; 6.476]
	RMC ^2^	4.236 ± 0.406	[3.244; 5.228]
	Total	4.463 ± 0.340	[3.716; 5.211]
**Grade I (*n* = 16)**	LMC ^1^	4.520 ± 0.330	[3.714; 5.326]
	RMC ^2^	3.038 ± 0.426	[2.056; 4.021]
	Total	3.687 ± 0.331	[2.982; 4.393]
**Grade II (*n* = 6)**	LMC ^1^	3.520 ± 0.001	-
	RMC ^2^	3.264 ± 0.068	[3.076; 3.452]
	Total	3.307 ± 0.070	[3.126; 3.488]
**Grade III (*n* = 6)**	LMC ^1^	2.973 ± 0.327	[1.933; 4.012]
	RMC ^2^	2.388 ± 0.426	[-3.019; 7.794]
	Total	2.778 ± 0.265 *	[2.098; 3.458]

^1^ Left mammary chain; ^2^ Right mammary chain; * *p* < 0.05.

**Table 6:** GNPs characterization over the consecutive synthesis steps. Size and PdI are represented as Mean ± SD (*n* = 3), with the size being represented by the most representative peak in terms of intensity %. The maximum absorbance peak is represented as the single value detected by the equipment ± the equipment uncertainty.

	Main Peak (nm)	PdI	Maximum Absorbance Peak (nm)
**Core GNPs**	252.4 ± 9.3	0.734 ± 0.025	899 ± 1
**HAOA-coated GNPs**	334.4 ± 40.4 *	0.637 ± 0.089	A broad band
**EGF-conjugated GNPs**	191.6 ± 17.3 ^##^	0.384 ± 0.024 ***^, ##^	823 ± 1

The statistical analysis results are represented as * *p* < 0.05; *** *p* <0.001 when comparing with the core GNPs and ^##^ *p* < 0.01 when comparing with the hyaluronic and oleic acid (HAOA)-coated GNPs.

**Table 7:** Hemolytic activity of Core GNPs and EGF-conjugated GNPs. The data is represented as Mean ± SD, *n* = 3.

GNPs Conc. (mg/mL)	Hemolysis (%) (Mean ± SD)	
	Core GNPs	EGF-Conjugated GNPs
0.7	0.0 ± 0.1	2.0 ± 0.2
3.5 × 10^−1^	0.0 ± 0.1	0.2 ± 0.2
17.5 × 10^−2^	0.0 ± 0.1	0.0 ± 0.3
87.5 × 10^−3^	0.0 ± 0.2	0.0 ± 0.3
43.8 × 10^−3^	0.0 ± 0.1	0.0 ± 0.2
21.9 × 10^−3^	0.0 ± 0.1	0.0 ± 0.1
10.9 × 10^−3^	0.0 ± 0.1	0.0 ± 0.2
5.5 × 10^−3^	0.0 ± 0.2	0.0 ± 0.1
2.7 × 10^−3^	0.0 ± 0.1	0.0 ± 0.1
1.4 × 10^−3^	0.0 ± 0.1	0.0 ± 0.1
0.7 × 10^−3^	0.0 ± 0.2	0.0 ± 0.1
0.3 × 10^−3^	0.0 ± 0.1	0.0 ± 0.1

**Table 8:** Histological evaluation of mammary tumors (chemically-induced DMBA) regarding several parameters classified with a score between 0 and 3 in a group without treatment (control group) and in a group where EGF-conjugated GNPs combined with laser irradiation (treatment group) were used.

Group	Tumor Grade	Necrosis	Hemorrhage	Stromal Reaction	Inflammatory Infiltrates
**Control group** **(*n* > 10)**	I	0.19 ± 0.09	0.19 ± 0.09	1.91 ± 0.12	1.86 ± 0.14
	II	1.25 ± 0.41	0.63 ± 0.38	2.25 ± 0.16	2.13 ± 0.30
	III	2.38 ± 0.42	1.13 ± 0.48	2.88 ± 0.13	2.88 ± 0.13
**Treatment Group** **(*n* = 10)**	I	1.00 ± 0.52 *	1.17 ± 0.48 *	2.67 ± 0.21 **	2.00 ± 0.37
	II	3.00 ± 0.01	2.50 ± 0.50	2.50 ± 0.50	3.00 ± 0.01
	III	1.50 ± 1.50	1.50 ± 0.50	3.00 ± 0.01	3.00 ± 0.01

Results are represented as Mean ± SEM and statistically significant differences are identified with * *p* < 0.05 and ** *p* < 0.01.

**Table 9:** Relevant morphological characteristics.

Necrosis	Hemorrhage	Stromal Reaction	Inflammatory Infiltrates
0–not present	0–not present	0–absent	0–absent
1–focal (10%)	1–focal (10%)	1–mild	1–mild
2–moderate (20–70%)	2–moderate (20–70%)	2–moderate	2–moderate
3–extensive (>80%)	3–extensive (>80%)	3–high	3–high

## Supplementary Materials

The following are available online at https://www.mdpi.com/1422-0067/21/24/9681/s1, Figure S1: GNPs’ size distribution by intensity (%) obtained by DLS.

## Abbreviations

5mC	5-methylcytosine
Abs	Absorbance
ALP	Alkaline Phosphatase
ALT	Alanine Transaminase
AST	Aspartate Transaminase
ATCC	American Type Culture Collection
BRCA1	Breast Cancer 1 gene
BRCA2	Breast Cancer 2 gene
BUN	Blood Urea Nitrogen
CI	Confidence Interval
DGAV	Directorate-General for Food and Veterinary
DLS	Dynamic Light Scattering
DMBA	7,12-dimethylbenzantracene
DMEM	Dulbecco’s Modified Eagle’s Medium
DMSO	Dimethyl Sulfoxide
DNA	Deoxyribonucleic acid
EDTA	Ethylenediamine tetraacetic acid
EGF	Epidermal Growth Factor
EGF-conjugated GNPs	Gold Nanoparticles coated with a combination of Hyaluronic and Oleic Acids to which EGF was added
EGFR	Epidermal Growth Factor Receptor
ER	Estrogen Receptor
GLOBOCAN	Global Cancer Observatory Reports
GNPs	Gold Nanoparticles
H&E	Hematoxylin and Eosin stain
HA	Hyaluronic acid
HAOA coating	Coating of Hyaluronic and Oleic Acids
HAOA-coated GNPs	Gold Nanoparticles coated with a combination of Hyaluronic and Oleic Acids
HCT	Hematocrit
HER2	Human Epidermal Growth Factor Receptor 2
LMC	Left Mammary Chain
MCF-7 cells	Michigan Cancer Foundation-7 Cells
MCH	Mean Corpuscular Hemoglobin
MCHC	Mean Corpuscular Hemoglobin Concentration
MCV	Mean Corpuscular Volume
MDA-MB-231 cells	M. D. Anderson Cancer Center- MB-231 Cells
microRNAs	Micro-Ribonucleic Acid
MTT	3-(4,5-Dimethylthiazol-2-yl)-2,5-Diphenyltetrazolium Bromide
NGS	Nottingham grading System
NIR	Near-infrared
NPs	Nanoparticles
OA	Oleic Acid
OD	Optical Density
ORBEA	Animal Welfare and Ethics Body
PBS	Phosphate Buffered Saline
PdI	Polydispersity Index
PR	Progesterone Receptor
PTT	Photothermal Therapy
RMC	Right Mammary Chain
RT	Room Temperature
SD	Standard Deviation
SEM	Standard Error of the Mean
SPR	Surface Plasmon Resonance
TEB	Terminal End Buds
TEM	Transmission Electron Microscopy
TE Buffer	Tris + EDTA Buffer
