# Supplementary material for: A Step Forward in Breast Cancer Research: From a Natural-Like Experimental Model to a Preliminary Photothermal Approach
Source: Int J Mol Sci. 2020 Dec 18;21(24):9681. doi: 10.3390/ijms21249681 (PMC7765974; doi:10.3390/ijms21249681)
Supplement: Supplementary file 1 [file ijms-21-09681-s001.pdf]

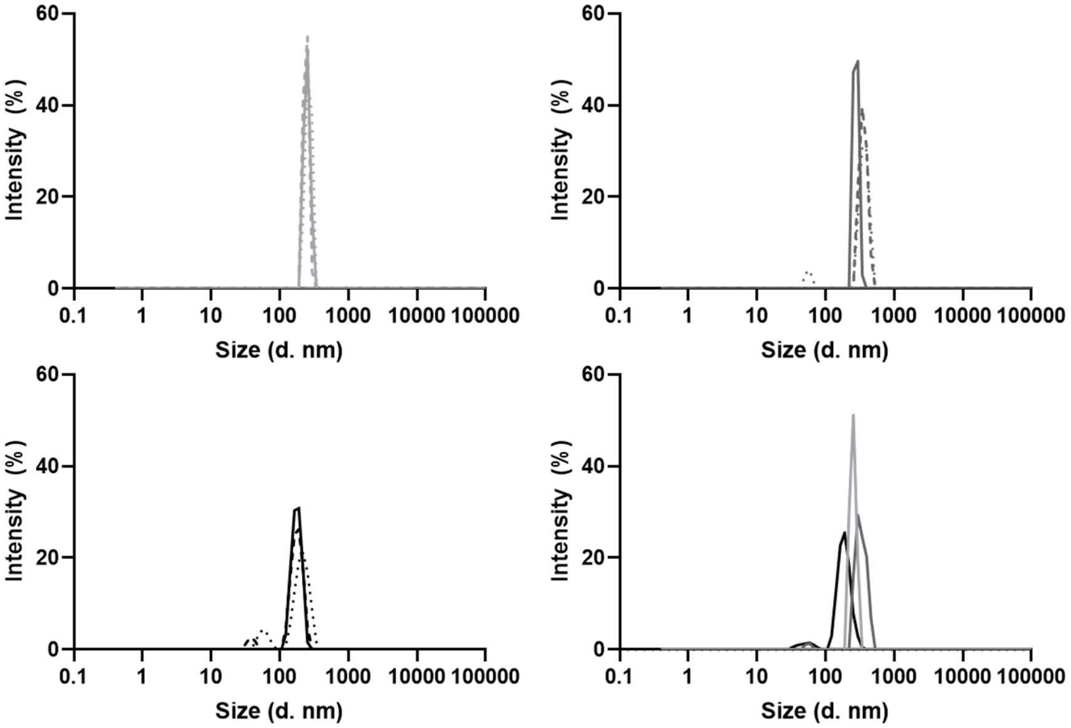

Figure S1: GNPs' size distribution by intensity (%) obtained by DLS. Three curves (full, dotted and dashed), each one of them corresponding to individual measures, are shown for each type of GNPs: Core GNPs (light grey), HAOA-coated GNPs (dark grey) and EGF-conjugated GNPs (black). Additionally, it is represented (bottom right corner) the average curve of each sample for comparison of the GNPs' types.
